# A Practical Work on the Diseases of the Eye, and Their Treatment, Medically, Topically, and by Operation

**Published:** 1841-01

**Authors:** 


### ART. III.

*A Practical Work on the Diseases of the Eye, and their Treatment, Medically, Topically, and by Operation.* By FREDERICK TYRRELL, Senior Surgeon to the Royal London Ophthalmic Hospital; Surgeon to St. Thomas's Hospital; Professor of Anatomy and Surgery at the Royal College of Surgeons in London, &c. Two Volumes; *with Plates.—London,* 1840. 8vo, pp. 533, 566.

IN an introduction extending to considerable length, Mr. Tyrrell summarily states the general pathological and therapeutical views brought forward in the body of his work. The general treatment of ophthalmic diseases, according to Mr. Tyrrell, should have for its object, the regulation of the most important secretions, the regulation of the general power, and the correction of local morbid action.

"Regulation of the general power is, in my opinion, of the highest importance, for I am satisfied that a salutary and curative process cannot be established or maintained whilst the general power is, on the one hand, in excess, or *above par;* or, on the other hand, when it is very deficient or much *below par.*" (p. xxxvii.)

The means enumerated for effecting a diminution of power when it is in excess or *above par* are, principally, spare diet, free action upon the principal secretions, quietude, and abstraction of blood. In regard to the last, our author says (p. xxxviii.) that it "should be resorted to only in urgent and hazardous cases, and then it should be employed cautiously. I consider," he continues, "the general abstraction of blood to be unnecessary and improper, unless the pulse evinces a degree of *resistance* or *incompressibility*, besides an unusual degree of fulness or quickness." It seems to us that Mr. Tyrrell is too much afraid of venesection, and that he is too exclusive in his indications for it. Fully appreciating the importance of *incompressibility* of the pulse as an indication—a point so ably insisted on by Mr. Wardrop—we would have it remembered that, as this distinguished ophthalmological writer remarks, "there are other qualities perceptible in the radial pulse which also indicate the propriety of bloodletting," whether "taken singly or combined with other symptoms, more particularly local pain, hot skin, and white tongue." In the case of the eye, circumorbital pain and redness, it may be mentioned, are, even when taken singly, an excellent criterion; for whenever these are present, the blood is buffy and the relief from venesection striking. Mr. Tyrrell mentions, as an exception to his rule, the slow, laboured, and compressible pulse which usually exists when there is cerebral mischief or disease. In this case the abstraction of blood, "by relieving excess of cerebral vascular action or pressure, favours the return of a proper degree of nervous power." We have repeatedly seen the same thing effected in the state of depression depending merely on the severity and continuance of circumorbital pain. The "decided and rapid relief of urgent local symptoms" we consider an object of so much consequence that it is to be sought for, even at the sacrifice of slightly protracting the cure. But we deny that the prostration of strength leading to this effect, much less to obstinacy or difficulty of cure, is necessarily produced by that extent of bloodletting which, along with the auxiliary remedies, usually procures the degree of relief above mentioned. It appears to us that Mr. Tyrrell in justly reprobating one extreme falls into the opposite. The following observations of Mr. Lawrence appear to us so completely to meet Mr. Tyrrell's partial objections to bloodletting, that we cannot refrain from quoting them, long as they are, particularly as we are persuaded that practice founded on such principles is much less likely in the hands of the many to produce mischief than the practice founded on the opposite principles which Mr. Tyrrell would inculcate:

"I know of no criterion," says Mr. Lawrence, "by which we can determine in all cases, whether general or local bleeding should be employed, If inflammatory fever coexist with the local disorder, we should abstract blood from the system; but we cannot say conversely that if such fever be absent we ought to be content with local depletion; an inflammation of the eye, for example, may require free general depletion, although it should not be attended with fever. (p. 105.) ............ I never," he continues, "saw a person injured by a single large bleeding performed for an active inflammation; while generally the strength is completely restored in twelve or twenty-four hours, even after bleeding to syncope. As the restoration of the digestive functions and the secretions diminishes the symptoms, where inflammatory fever is present, they who are afraid of weakening by loss of blood recommend in preference aperient and diaphoretic medicines. If you examine the history of cases treated in this way, you find that two, three, or more days are employed in those indirect attempts. Purgatives are given which do not operate; diaphoretics are tried which bring on no discharge from the skin; the local inflammation increases; the general disturbance is aggravated, until the fever comes to an end, when the secretions and digestive functions are consequently restored. Abstraction of blood to a proper amount accomplishes the desired objects at once. When you have thus removed the load that oppresses the system, the suspended secretions are restored, evacuation of the bowels takes place speedily, and the patient breaks out into a profuse perspiration. Thus the sufferings of the patient are materially abridged, while the duration of the local disturbance is shortened; the latter being a very important point in the case of the eye." (p. 106.) (*Treatise on the Diseases of the Eye.*)

The means of promoting and maintaining power are principally diet, stimuli, and tonics. Mr. Tyrrell's remarks on tonics are good:

"The selection of the tonic should be regulated by the character of the debility and the condition of the patient: thus when the cause of the debility has been loss of blood, or when the patient is very pallid, and has cold extremities, and small, quick, but feeble pulse, the preparations of steel or zinc will, usually, prove most efficacious: when the patient has been exhausted by severe or protracted febrile disease, by diarrhœa, or by want of proper nourishment, the preparations of bark frequently promote the desired purpose better than the mineral preparations; in those of feeble power and scrofulous diathesis, or in such as have been suffering from specific taint, sarsaparilla, with minute doses of iodine or mercury, generally effect most good: and in such as evince unusual nervous susceptibility with feeble power, the addition of ammonia, valerian, castor, &c., to some of the tonics which I have mentioned, is, frequently, very serviceable." (p. xlii.)

In discussing "the correction of local morbid action by the influence of alterative medicines," Mr. Tyrrell considers at some length the use of mercury, which he justly characterizes as "perhaps, the most useful as well as the most powerful remedy in many of the most important ophthalmic diseases." Much, however, he remarks, depends on the mode of its administration. He dissents from the opinion that mercury acts most beneficially whilst the patient is kept upon a very abstemious diet, and lays it down as "a general rule, that whenever it is necessary to give mercury for the continuance of a few weeks, that it is essential to promote and maintain power at the same time." (p. xlv.)

Here it may be remarked that Mr. Tyrrell's doctrine about "power" strongly reminds us of the Brunonian theory. It appears to be equally founded on the supposition that the body is a sort of machine regulated by the mere abstraction or addition of stimuli. In urging so much as he does the promoting and maintaining of "power," Mr. Tyrrell seems not to reflect that the patient often cannot bear generous diet, because the stomach partakes of the general debility. Nor does he take into account, in laying down his indications and counter-indications for depletion at p. xxix., correct as they may be in the main, that cases do occur in which, notwithstanding the existence of debility, considerable depletion is the only means of arresting the destructive progress of inflammation.

Besides what has been just noticed, Mr. Tyrrell considers, in his introduction, several topics of great technical importance, such as the mode of examining the eye and the application of local remedies. On the latter topic we subjoin a few extracts and remarks.

Mr. Tyrrell bears testimony to the advantage of employing warmth and moisture by fomentations and steam. The latter, he says, "is perhaps preferable to the former when the affected organ is excessively tender." The mode of application recommended is to invert a large funnel over a jug containing the heated fluid, when "the steam which escapes from the narrow end of the tube can be received against the palpebræ, at a distance agreeable to the patient."

*Lotions*, Mr. Tyrrell recommends to be applied with a piece of soft linen to the surfaces of the eyelids gently closed; when sufficient of the fluid will penetrate the palpebral aperture, to effect the desired purpose. He properly objects (page xlviii.) "to the use of eyeglass, syringe, or other such method of applying lotions," telling us at the same time he has seen much mischief from them. We consider the eyeglass a most absurd implement. In applying drops to the eye, "the surgeon should depress, and slightly evert the inferior eyelid, by pressing on the integument of the cheek, and then pass the loaded brush between the lid and the surface of the globe." (p. xlix.) Though in this case the fluid quickly passes over the surface of the conjunctiva of the lower eyelid and lower part of the eyeball, it does not affect the conjunctiva of the upper eyelid and upper part of the eyeball so completely as is desirable in many cases, because even the small quantity which, by the mode of application above recommended, makes its way between the upper eyelid and eyeball, is quickly washed away by the torrent of tears which follows the operation. We have, therefore, frequently found it of the greatest service either to evert the upper eyelid and pencil its inner surface, or, drawing it from its contact with the surface of the eyeball, allow the drops to flow freely underneath. We can also affirm from multiplied and comparative experience, that ointments introduced under the upper eyelid and diffused over the eye by rubbing the eyelid over the eyeball with the finger often proves of great service, though we infer that Mr. Tyrrell objects to this mode of employing ointments when he says (p. li.) that it is not "the intention of the application" that it should "penetrate in quantity between the palpebræ to the surface of the eye." And as a further objection to letting an ointment penetrate between the eyelids to the surface of the eye, he remarks that "the purest grease put into the eye produces irritation by acting as an extraneous matter." (p. li.) True; and so does even pure water, much more, stimulating drops. And if it be admissible and necessary to apply drops within the lower eyelid, it is so also to apply them within the upper eyelid; and if drops, ointments too: for the latter may be frequently substituted with advantage for the former.

Mr. Tyrrell approves of dry warmth, maintained by applying over the eye small bags lightly filled with chamomile flowers or bran or the farina of beans, and mixed or not, according to circumstances, with narcotics or aromatics. These vegetable bags, *Kräuterkissen*, are much used in Germany. Dry warmth thus applied, Mr. Tyrrell remarks, "affords great relief in some cases." (p. l.)

Mr. Tyrrell finds the outer surfaces of the eyelids or the cheek a little below the inferior palpebræ, the best places in which to apply leeches. He observes (page li.) that "they have very little effect in relieving the vessels of the conjunctiva, when placed upon the temple." He disapproves of the application of leeches to the conjunctiva itself, on the just ground that the point which has been bitten is left elevated and irregular, and thus irritates like a foreign body. We are not, however, prepared to agree with him that the same effect is produced by scarification of the conjunctiva in ordinary cases in a degree so great as to counterbalance its advantages, and therefore to justify the condemnation of it. When leeches cannot be procured, or when their use is forbidden, as when from idiosyncracy they excite inflammation of the skin, the angular vein, if well developed, may, Mr. Tyrrell says, sometimes be opened with advantage; otherwise he recommends cupping on the temple, or behind the ear, a little below the mastoid process.

As counter-irritants, Mr. Tyrrell generally employs "liniment of ammonia, mustard-plaster, tartar-emetic plaster, blister, and issue." Blisters are, according to Mr. Tyrrell's experience, more efficacious when frequently repeated than when kept open by irritating ointment. Amongst other objections to *issue or seton*, Mr. Tyrrell mentions that he has known instances of the indelible scar, which results being detrimental to the prospects of that class of persons destined for servitude by preventing them being accepted as servants. Mr. Tyrrell concludes his introduction with remarks on the relief obtained by position in acute diseases.

"I have often heard the sufferer observe, that he had been sitting up in bed the greater part of the night, and that he had been easy, or his pain had been very much lessened whilst he maintained such a position; but that his symptoms became aggravated as soon as he resumed the recumbent posture." (p. lviii.)

We believe the relief here mentioned is as much owing to the circumstance of the patient's head being cooler while sitting up in bed as to position. The painful heat attending conjunctivitis is in this way relieved; but above all, the circumorbital or temporal pain attending catarrho-rheumatic ophthalmia, iritis, &c., the peculiarity constantly remarked in regard to which is that it becomes worse as the patient gets warm in bed. Persons who have suffered from rheumatic toothach must have remarked the same thing, and, besides the sitting up in bed, how much a mouthful of cold water mitigates the pain.

In entering on the special part of our theme, we must premise that Mr. Tyrrell follows an anatomical arrangement, successively taking up the different parts of the eyeball from the conjunctiva inwards. By this plan the diseases of the ocular appendages would have come in last; but, "in order to prevent a great inequality in the size of the volumes," they are placed at the end of the first volume, "in preference to dividing the subject of AMAUROSIS, which must have been done had the arrangement originally intended been followed." The consideration of the diseases of each part is prefaced by a short sketch of its anatomy and physiology.

*Anatomy of the Conjunctiva.* The general disposition of the conjunctiva seems to us to be somewhat clumsily, and, even not very accurately described. The structure of the membrane, we are told (p. 4), appears to be chiefly cellular; and, although Mr. Tyrrell believes it to be properly classed with the mucous membranes, he says, "it does not exhibit in the natural state a villous appearance, which however becomes apparent in some states of disease." A villous, or rather papillary appearance on the palpebral conjunctiva, in the healthy state, may be perceived with the naked eye, and quite distinctly if a magnifying glass be used. Mr. Tyrrell passes over without notice the epithelium, with which the conjunctiva is invested.

The following statement sounds oddly, until we come to learn that Mr. Tyrrell means by *organization* the possession of blood-vessels, nerves, and absorbents: "The conjunctiva, in the natural state, exhibits but slight traces of its organization." (p. 4.) And the reason it does so, he informs us, is, that it is "chiefly supplied by serous vessels, very few being capable of circulating the red particles." Modern physiology assures us that "serous vessels" and vessels incapable "of circulating the red particles" are mere things of the imagination. The red globules follow one another perhaps in single or in double series in the conjunctival vessels, which, accordingly, are still invisible; but when more globules get alongside of each other then the vessels are seen.

*Morbid Conditions of the Conjunctiva.* In describing the diseases of the conjunctiva, Mr. Tyrrell says (p. 9) he shall confine himself to "such divisions as lead to practical good, and render unnecessary technical minuteness;" and, in conformity with this resolve, he admits the following varieties of inflammation of the conjunctiva: to which it may be remarked, in passing, he confines the term "Ophthalmia"—"Simple ophthalmia; pustular ophthalmia; catarrhal ophthalmia; purulent ophthalmia; chronic ophthalmia, not as a result of acute disease, but originating in chronic form; strumous or scrofulous ophthalmia; and exanthematous ophthalmia. The last two are modifications of most of the above varieties."

*Simple Acute Ophthalmia.* The simplest example of this would be traumatic conjunctivitis; but as the inflammation of the conjunctiva, excited by injury, may, according to the age and state of health of the patient, be puro-mucous, pustular, or phlyctenular, it is obvious that simple acute ophthalmia, or, as it is otherwise designated, "simple taraxis," or the "milder form of external ophthalmia," must frequently vary in character. Accordingly, we find that Dr. Mackenzie does not give it a separate place in his list at all, but describes it as a milder form of catarrhal ophthalmia; while Mr. Tyrrell has brought together, under this head, examples of different kinds of disease, or, as he considers them, modifications of simple acute ophthalmia, depending on disorder of stomach, liver, skin, or uterus, or on general debility.

The following is the manner Mr. Tyrrell arranges his descriptions: 1, the definition of the disease; 2, the synonymes; 3, the local symptoms (local subjective symptoms); 4, appearances (objective symptoms); 5, constitutional symptoms; 6, direct causes; 7, predisposing causes; 8, persons liable to; 9, modifications; 10, treatment.

We highly approve of systematic arrangements of this kind; but we find that too strict an adherence to his plan has occasionally led Mr. Tyrrell into difficulties. Thus, in describing the *local symptoms* of entropium (vol. i. p. 438,) he successively enumerates "a constant state of irritation, as if from the presence of an extraneous body;" pain "especially experienced on moving the globe;" lacrymation, incapacity of directing the vision to any useful purpose and intolerance of bright light—as if these were at all pathognomic, and as if the nature of the disorder could not be at once recognized on inspection and the local symptoms inferred.

Mr. Tyrrell lays down the treatment of simple acute ophthalmia very judiciously, and well observes, that "it must be recollected that important functional derangement is very frequently a predisposing cause of ophthalmia, and rarely the direct cause; but, when once the disease is excited, that which has predisposed modifies it, and continues to have a material influence over it." (p. 13). Fully appreciating the propriety of Mr. Tyrrell's treatment, we think that, in relating the illustrative cases, he exhibits too great an inclination to the *post hoc propter hoc* mode of reasoning; inasmuch as he appears to refer all to the last, and to lose sight of the influence of the treatment previously adopted.

Mr. Tyrrell describes very well (p. 23) those cases, so frequently met with in consultations, of obstinate ophthalmia resulting from too long a continuance of depletion; or, perhaps, when this has been properly enough regulated, from too long a delay in giving strengthening diet and medicines. But, in dwelling so much on the rapidity of cure consequent on their exhibition, and attributing so much to them, he seems to forget that here, also, the way was prepared by the previous depletion. It may be remarked, that, in some cases, a cure of the local complaint is effected with an equal rapidity merely by one or two applications of some stimulant to the part. He relates several interesting cases of "simple ophthalmia," illustrating the utility of change of air and strengthening treatment in persons labouring under a peculiar condition of debility, independent of any important functional derangement (p. 28); in other words, the *Cachexia Londinensis* of Sir James Clark. In this state of health sometimes the eye, sometimes another organ may suffer; Mr. Tyrrell does not lose sight of this, but very properly collates the disease of the eye with analogous cases in general surgery, curable by the same means, such as "inflammation of the ligaments or synovial membrane of a joint, of the mucous membrane of the urethra, of the vagina, of the nose," &c. &c. (p. 35.)

*Pustular Ophthalmia.* Mr. Tyrrell mentions that the pustules in this disease occur on the sclerotic conjunctiva, at a short distance from the cornea, at the margin of the cornea, and on the cornea itself. Taking such an indiscriminate view of pustular ophthalmia, it is no wonder he should have sometimes met with rapid cure from the application of a solution of nitrate of silver, but more frequently failure. We believe it is not the remedy which is so "uncertain" as his diagnosis; for if he had used the nitrate of silver in solution or ointment, or indeed any other analogous application, as the red precipitate ointment, in those cases only in which the aphtha or pustule was on the sclerotic conjunctiva a tenth or twentieth of an inch from the margin of the cornea, he would seldom have found the remedy fail. When the pustule is on the cornea or its margin, the curability of the complaint is entirely different. The pustular ophthalmia, so called when the pustules are over the sclerotica, being, as Dr. Mackenzie well points out (p. 430), "less dangerous and more tractable than the phlyctenular (or scrofulous ophthalmia, properly so called,) into which, or into the scrofulo-catarrhal, it sometimes has a tendency to pass." Mr. Tyrrell has not only mixed up these in his description of "pustular ophthalmia," but also certain forms of exanthematous ophthalmia. Pustular ophthalmia, Mr. Tyrrell says, is occasionally combined with catarrho-rheumatic ophthalmia. It is true that, in catarrho-rheumatic ophthalmia, ulceration of the cornea is very apt to succeed a phlyctenula or onyx; but we do not find pustular ophthalmia, as commonly and properly understood, combined with catarrho-rheumatic. And it is to be remarked, that the subjects of the former affection are for the most part children or young adults, whereas the subjects of the latter are generally old persons. We cannot but consider that, in his chapter on pustular ophthalmia, our author makes one of the most simple matters in the whole range of the diseases of the eye confused and difficult.

*Catarrhal Ophthalmia.* What Mr. Tyrrell describes under this head is the severer form of the disease, as given by Dr. Mackenzie. In describing the evening exacerbation, Mr. Tyrrell mentions that the patient "usually experiences *headach and pains in the orbit.*" Orbital pain, aggravated at night, is a very important symptom in all inflammations of the eye; because, as we have already mentioned, its existence is in general a very good indication of the necessity for venesection. Orbital pain should therefore always be carefully distinguished from mere headach, and especially from that pain and sense of weight about the frontal sinuses and antrum, met with in severe cases of catarrh. It is this kind of headach which may be found in catarrhal ophthalmia, not pain around the orbit or in the temples. Where the latter pain exists, it will be found that to the inflammation of the conjunctiva there is joined inflammation of the sclerotica or iris.

It is true that "the catarrhal disease is usually very tractable, and does not require severe remedies to subdue it" (p. 48); but it ought to be borne in mind that it is not so always, nor everywhere. It is a disease which ought to be got speedily under, on account of its great tendency to leave the conjunctiva in a thickened, if not granular state; a state from which it does not readily recover. We believe that, in the severer cases, well-timed abstraction of blood by leeches or venesection, and local stimulants, will not fail the practitioner. We agree with Mr. Tyrrell in his objections (p. 46) to the employment of cold in catarrhal ophthalmia, but we cannot subscribe to his indiscriminate objections to strong stimulating applications, which, judiciously employed, we consider of the greatest value.

*Purulent Ophthalmia.* Mr. Tyrrell considers Egyptian ophthalmia, gonorrhœal ophthalmia, and ophthalmia neonatorum, as "virtually the same in all instances; only modified by circumstances of age, climate, mode of origin, &c." And although he describes, under different heads, the purulent ophthalmia of the adult, and that which occurs in the infant, it is "solely because, from the conditions of the patients, some difference in treatment is requisite." This is, after all, just doing what other authors do.

Mr. Tyrrell's plan of treating chemosis by incisions in the conjunctiva, radiating from the cornea, forms the leading feature of this chapter; but, as we touched upon this subject in our last Number, we shall not here return to it.

*Purulent Ophthalmia in the Infant.* There is nothing in this chapter calling for particular notice. It may merely be mentioned that Mr. Tyrrell believes the causes of the diseases to be threefold: First, and most commonly, leucorrhœa in the mother; secondly, less frequently, "a purulent secretion from the urethra of the mother (gonorrhœa);" thirdly, exposure to cold and damp. He thus omits one of the most frequent causes; viz., the intrusion of soap, spirits, &c. into the infant's eyes.

*Chronic Inflammation of the Conjunctiva, following Purulent and Catarrhal Ophthalmia.* In this chapter, what is called *granular conjunctiva*, or the *granulated eyelid* is considered. At p. 122, Mr. Tyrrell mentions the sclerotic and even the corneal conjunctiva as presenting the granular state; but this is an erroneous view of the matter. The appearances to which Mr. Tyrrell alludes, as being presented by the ocular conjunctiva, are not of the same nature; for the anatomical reason, that that part of the conjunctiva does not possess a papillary or villous structure, similar to that which, in the palpebral conjunctiva, forms the peculiar seat of the granular prominences.

Dr. Mackenzie correctly says that the ocular conjunctiva "may present a red and swollen appearance, but is not really granular" (p. 553), that is, presenting hypertrophied papillæ. The granular appearance sometimes observed on the cornea is owing rather to real granulations.

Mr. Tyrrell's account of the varieties of granulations on the palpebral conjunctiva is short, but sufficiently comprehensive and correct for practical purposes. "The villi are red, firm, and elastic, like the granulations of a healthy ulcer; or they are soft and flaccid: or small, hard, and of a light colour. Where the first or last varieties exist, the power of the patient is seldom much impaired; but when the second is found, the constitutional vigour of the patient is generally feeble." (p. 131.)

In our last Number we slightly touched upon the circumstance, that because the sclerotic conjunctiva may have become free from redness, it is not to be supposed, as Dr. Mackenzie remarks (p. 376), that the inflammation is completely subdued, for the palpebral conjunctiva may still remain in a morbid state. "The inside of the eyelids, and especially of the upper, ought daily to be inspected." This point is very well stated by Mr. Tyrrell:

"The heading of the section shows that I consider the disease which I have described as the result of purulent and catarrhal ophthalmia; but I deem it important to explain how, and why it occurs. I have shown that these diseases commence in the palpebral division of the conjunctiva, and from thence extend to the ocular portion. They disappear in the contrary order; leaving, first, the ocular part of the membrane, or that in which they appear last, and linger in the palpebral portion of the tunic, or that in which they first appeared; and, in this division of the membrane, the morbid action may remain in so trifling a degree as to escape the observation of the careless practitioner, who may be satisfied with the perfect restoration of vision, independently of slight occasional interruption, from a collection of superabundant secretion. In order to prevent the occurrence of the chronic affection, the palpebral conjunctiva should be carefully examined when the acute disease appears to have been completely subdued, and if the membrane of the eyelid has not perfectly recovered its natural aspect, the remedies should be continued until all morbid appearance be subdued. The examination should extend to the conjunctiva of both eyelids." (p. 124.)

With reference to the *treatment*, Mr. Tyrrell recognizes two stages of the diseases. The first stage he "would limit to the period before which the cornea becomes nebulous and vascular, or that in which the palpebral conjunctiva only presents a morbid aspect." In the second stage, the disease has produced a nebulous and vascular state of the cornea. The first stage, Mr. Tyrrell says, "can usually be subdued with very moderate care." The second "requires much more care and patience for its relief." In the treatment of the first stage, Mr. Tyrrell properly recommends good diet, tonics, and protection from the changes of weather, &c. Locally, he employs counter-irritation and stimulants. In regard to the use of the latter, we have found from much experience that it is best that they should be pretty strong, and applied, whether ointment or liquid, directly to the affected surface once a day; bedtime is the most convenient: we have also found it advantageous occasionally to intermit their use.

Mr. Tyrrell says (p. 130), "The treatment should be persevered in until the conjunctiva of the eyelids has regained its *natural appearance*, which it will not do till some time after the morbid secretions have disappeared." We wish we could say our experience bears out Mr. Tyrrell in this statement; we fear that it will be found that, even under the most favorable circumstances, and after the mildest form of disease, the *natural appearance* of the conjunctiva is not so readily regained. In the second stage, Mr. Tyrrell recommends the same general remedies as the first; but the local means proposed for it, he says, "will hardly be found sufficient." He thus expresses the principle of the local treatment he has found most effective: "Whenever there was evidence of congestion, the vessels were relieved by the application of the leech; and when this congestion had subsided, the contraction of the vessels was promoted, and the action of the absorbents excited, by applications having astringent and stimulating proportions." (p. 134.) He has occasionally substituted scarification for the leech, but does not consider the former much preferable to the latter, "excepting that it is more rapid in its effects, and is not likely to create extravasation or swelling, which the leech is apt to do." It has too often happened, that, in the local treatment, caustics have been sadly abused. On the whole, the plan of treating granular conjunctiva, recommended by Mr. Tyrrell, is what is now most resorted to, or rather what is now considered allowable.

*Simple Chronic Ophthalmia.* The disease which Mr. Tyrrell designates by this name affects chiefly adults, whose occupation exposes them to cold and damp, especially if addicted to intemperance. The disease, again, which he describes at p. 489, under the head of "tinea ciliaris," "usually occurs in children, and very rarely commences after the age of puberty. I have known it to arise in the adult." (p. 491.) Dr. Mackenzie appears to comprehend both under the name of "inflammation of the edges of the eyelids, or ophthalmia tarsi;" though his description bears most upon the disease occurring in youth. In a practical point of view, the separate consideration of the two forms of disease is to be preferred. This section of Mr. Tyrrell's work is excellent.

*Scrofulous Ophthalmia.* Mr. Tyrrell does not consider that there is any inflammation of the conjunctiva peculiar to scrofulous persons, but that all are occasionally modified by the scrofulous constitution, and suggests that the phlyctenular affections of the mucous membrane appear to be peculiarly scrofulous, merely on account of their greater frequency. The general appearances or physiognomy of the disease is very well delineated, only the symptoms are said to be aggravated at night, whereas the peculiarity of scrofulous ophthalmia is, that the symptoms remit towards evening. Our author gives a good account of the disordered state of health with which scrofulous ophthalmia is so generally accompanied, especially of the disorder of the digestive organs, already so well described by the late Dr. T. J. Todd in the Cyclopædia of Medicine, under the name of strumous dyspepsia.

Under the head of *Causes*, Mr. Tyrrell very well remarks:

"The conjunctival affection may, and probably does, in a large majority of these cases, result from the ordinary causes, which I have enumerated under the subjects of simple and pustular ophthalmia; but, when once induced, it becomes influenced, promoted, and sustained by some functional disorder, combined with the peculiarity of constitution." (p 155.)

Mr. Tyrrell very properly places his main reliance in the cure of this disease on constitutional treatment; but he says nothing of emetics, which generally prove so valuable at the commencement. The repeated application of small blisters, simple tepid water, or decoction of poppy heads or chamomile flowers as an eye-water, and occasionally a few leeches to the eyelids, make up his local treatment. Concerning the application of cold, he remarks, that it is sometimes grateful to the sensation when first used, but seldom fails to augment the suffering if it be continued. Mr. Tyrrell does not notice, among his remedies for scrofulous ophthalmia, conium, hyosciamus, and belladonna; which, especially the latter, are of so much service in subduing the intolerance of light.

*Chronic Scrofulous Ophthalmia.* The disease described under this name appears to be scrofulo-catarrhal ophthalmia, become chronic and fixed in the conjunctiva corneæ. The treatment recommended by Mr. Tyrrell contrasts advantageously with the frittering and exhausting—the indefinite and inefficient treatment too often employed; still, we think, he makes rather too much of it: harping about "power," "function," and the like. We consider general treatment of paramount importance; but our own experience convinces us that in a chronic disease of the conjunctiva corneæ, such as this, some local stimulant, properly applied, and that even two or three times only, is capable of producing in a few days, as striking an effect as that mentioned by Mr. Tyrrell, arising from the restoration of uterine function, or months of general treatment alone. Of course, we do not confine ourselves to the mere local treatment, but endeavour to improve the health, and thus prevent the tendency to relapse.

There being nothing to arrest attention in the chapter on "exanthematous ophthalmia," we proceed to notice some of the organic diseases of the conjunctiva.

Mr. Tyrrell successively considers "Excrescences of the Conjunctiva," "Pterygium," which, he says, has generally a *conical* form; fræna, by which is meant symblepharon, though this or any other synonyme is not given. In regard to fræna, Mr. T. tells us that he is now convinced, after repeated trials, that operations for their prevention or removal are worse than useless. He describes, under the name of "Adipose tumours of the conjunctiva," what are commonly called *pingueculæ* or *pterygia pinguia*, though here Mr. Tyrrell again omits his practice of giving synonymes. "The tumour," he says, "consists of a deposit of fatty or adipose matter." Weller analyzed the matter, and assures us that it is not fatty, but rather a substance of a nature betwixt albumen and gelatine. Has Mr. Tyrrell ascertained that they are really fatty tumours, to warrant him to lay aside the conventional, though erroneous Latin names, and, by rendering them into English, to give them, as it were, a more literal force?

In speaking of injury of the eye from strong acids, Mr. Tyrrell fortunately does not manifest much individual knowledge of the very destructive effects, immediate or remote, sometimes observed. "After sulphuric acid," says Dr. Mackenzie (p. 226), "has been thrown into the eyes (a piece of diabolical malice), the effects of which I have repeatedly had occasion to witness, the conjunctiva almost appears scarred, being white, soft, and swollen. It afterwards peels off,* while the cornea

* As this is passing through the press, we observe that, in a communication made to rapidly becoming disorganized by infiltration of pus, ulceration, and sometimes sloughing, a raw surface is left both on the ball and on the inside of the lids, ready to unite and close the eye by an incurable and almost total symblepharon.....It is worthy of remark, that dangerous symptoms, as onyx and iritis, are apt to occur, in such cases, weeks from the receipt of the injury, and after the immediate effects have subsided."

Nothing very novel appears under the head of local injuries to the eye. That this organ can bear a great deal is proved by the strong stimulants frequently applied to it by way of treatment. Dr. Mackenzie quotes from Ammon's Zeitschrift the case of a man who had a drop of melted pitch fall directly on the cornea, where it stuck so fast that it could not be loosened until softened by the application of olive oil, when it quitted the eye without leaving any visible injury. And Mr. Tyrrell informs us, that he has "several times taken out, from beneath the palpebræ, portions of lead weighing many grains, which had evidently entered the eye in a fluid state, as they have been perfectly moulded to the surfaces of the globe and palpebræ, yet the conjunctiva has remained free from all injury, except slight inflammatory action." (p. 204.)

*Diseases of the Cornea.* The anatomical sketch which precedes the consideration of these is not characterized by much accuracy. Passing it over, and passing over for the present the diseases described by Mr. Tyrrell under the name of the "acute form of inflammation of the cornea, terminating in suppuration," when occurring idiopathically, and "inflammation of the cornea with vesication," as they appear to be identical with the diseases usually grouped under the name of catarrho-rheumatic ophthalmia, we come to Mr. Tyrrell's chapter on "Inflammation of the cornea, with deposition of earthy matter." The expressions employed in the description lead us to suppose that the earthy depositions were on the surface of the cornea, but it is not mentioned whether there was any ulceration underneath. The depositions he has met with, Mr. Tyrrell thinks, are the same as those described by "several eminent ophthalmic surgeons," as deposits from eye-waters containing acetate of lead. Deposits like wet chalk on ulcers of the cornea were described and represented many years ago by Mr. Wardrop in his Morbid Anatomy of the Eye, and Beer had mentioned his having observed that the use of lead lotions rendered the cornea opaque; but it was Dr. Jacob, we believe, who first particularly called attention to the circumstance, that if a solution of acetate of lead be applied to any part of the conjunctiva in an excoriated or ulcerated state, the acetate is decomposed, and a white precipitate is deposited, which adheres tenaciously to the conjunctiva, and, as the membrane heals, becomes fixed in the cicatrice. Dr. Jacob describes and delineates depositions in the palpebral conjunctiva, as well as on the cornea. He says (Dublin Hospital Reports, vol. v. p. 370,) "The opacity appears to be produced at once by a single application. I have seen it the day after a drop of solution of acetate of lead had been put into the eye by the British Association at Glasgow, the epidermic part of the conjunctiva corneæ, which is rendered opaque and peels off, is erroneously supposed to be a false membrane. The author, Dr. R. D. Thomson, does not seem to be aware of the difference in the chemical composition of the epidermic layer, and the proper substance of the cornea; and consequently that the reagents which render opaque the former have not the same effect on the latter.

mistake." He further says, "I do not think that I can state positively the precise condition of the ulcer which causes this deposit, but it does not take place in all cases where the acetate of lead is used." The most marked examples we remember to have seen were in cases of ulcer in catarrho-rheumatic ophthalmia. Mr. Tyrrell considers that the depositions in the cases observed by him were not from any metallic salt, though he does not deny that opacity may occasionally result from the adherence of an insoluble precipitate on an ulcerated surface. He adds, however, "I must confess that I have never seen a satisfactory case of the kind." Be this as it may, it is quite clear that the grounds on which Mr. Tyrrell would establish "Inflammation of the cornea, with deposition of earthy matter," as a distinct form of disease, are quite unsatisfactory.

*Corneitis* is the synonyme given by Mr. Tyrrell to "Inflammation of the cornea, with effusion of adhesive matter or fibrine." In the treatment of this affection, Mr. Tyrrel recommends mercury; which, considering that the "disease most frequently occurs in young persons of scrofulous habit and feeble power," must be cautiously administered. Besides the mercurial, some tonic, selected according to the circumstances already mentioned in regard to the exhibition of tonics generally, is judiciously recommended.

*Inflammation of the Cornea, terminating in Suppuration.* Mr. Tyrrell recognizes two forms of this, the acute and chronic. As a preliminary to his description of these, he enters into a learned disquisition on the meaning of the terms *Onyx* and *Hypopyon,* and quotes the authority of St. Ives to show that we now-a-days most perversely use the one word for the other.

Mr. Tyrrell's "chronic suppuration of the cornea" is the effect of inflammation occurring in exhausted states of the system. He mentions that he has seen it most frequently in children who have suffered from febrile disease, as measles, scarlatina, &c., and also in old persons after injury. We have seen it in a child in a state of great exhaustion after typhus fever, and we have seen it occur in both eyes in an old man who was subjected to the operation for cataract.

*Ulcers of the Cornea.* Of these Mr. Tyrrell describes three principal forms—the *healthy,* the *inflammatory,* and the *indolent.* The character of the healthy ulcer is, that its surface and circumference are hazy or opaque; the inflammatory ulcer may be recognized by the appearance of small vessels carrying red blood, in addition to the haziness or opacity observed in the healthy ulcer; the indolent ulcer appears clear and transparent, "merely a small dent presenting itself on a close inspection of the surface, as if a small portion had been cleanly excised by some sharp instrument." Mr. Tyrrell's healthy ulcer might, with perhaps more propriety, be called the healing ulcer, because it presents its peculiar characteristics as the inflammation which produced it is subsiding; and the inflammatory ulcer might be called active ulceration. When the inflammation is checked, the progress of the ulcer is in general also checked; the vessels running into it gradually diminish and retract, and in proportion to this the ulcer fills up and cicatrizes.

It is incorrect to say, in an unqualified manner, that "in the inflammatory ulcer the necessity for depletory measures is clearly indicated, and these must be steadily pursued until the visible vascularity of the ulcer is subdued." The state of the ophthalmia in general, of which the ulcer is an appearance, must alone regulate the depletion. Even after the ophthalmia is checked, the process of the disappearance of the vessels takes up some time. When it has commenced it is unnecessary to push it by depletion, this not being otherwise required. On the contrary, notwithstanding what Mr. Tyrrell says, we have seen the well-timed employment of a stimulating application hasten the retraction of the vessels and the cicatrization of the ulcer.

In speaking of the discoloration of the conjunctiva produced by the prolonged employment of nitrate of silver solution, Mr. Tyrrell says (p. 266), "it always commences at the lower part, where the membrane is reflected from the lid to the globe, and from this it gradually extends to the ocular and palpebral surfaces." The cause of this is, that the solution is always applied first to the lower part, and consequently in its most concentrated state, and is diffused in a diluted form over the rest of the conjunctiva. The wash should therefore be always put into the eye in such a manner, that it may, as we have already pointed out, be equally diffused under the upper and lower eyelids.

*Staphyloma Corneæ.* Mr. Tyrrell adopts the pathological views regarding the formation of staphyloma corneæ, at least in all their essential particulars, which were, we believe, first propounded by Mr. Wharton Jones. An abstract of Mr. Jones's paper, first published in the Medical Gazette, is given in our Fifth Volume, p. 612. To this, however, Mr. Tyrrell does not refer, and makes use of expressions in his description which would lead to the supposition, that, if the views alluded to have not hitherto been those universally received, they are his own.

"When the destruction of the cornea," says Mr. Tyrrell (p. 270), "has been so extensive as to allow of the protrusion of the iris, &c. which I have described as giving rise to the staphyloma, much may be done on the part of the surgeon to check the protrusion..... He should endeavour," Mr. Tyrrell says, "to excite the deposition of fibrine by the use of local stimuli, as the solutions of sulphate of zinc or nitrate of silver." This is treatment little likely to effect the desired object, and what was not to have been expected from one who appears in other cases so chary of local stimulants.

Escharotics have been principally used for reducing the size of partial staphylomata, and butter of antimony has been that most recommended. The apex of the staphylomatous projection has been the place to which it was usually applied. The following is the mode of treating partial staphyloma, practised by Mr. Tyrrell.

"Partial staphyloma," says Mr. Tyrrell, p. 273, "often exists under circumstances, which render its reduction a matter of importance or anxiety; as when there is sufficient of the healthy cornea left to enable the surgeon to form an artificial pupil beneath, or when the projection gives rise to much irritation or deformity. I have," Mr. Tyrrell says, "succeeded, in several instances, in effecting a reduction of partial staphyloma, by the careful application of nitrate of silver, or hydrate of potash, in substance. I have applied the escharotic first at the base of the projection, taking care not to injure the remaining sound cornea—the effect has been the separation of a small slough; but previous to such separation, a deposit of fibrine beneath, by which the deeper part has become more solid and strengthened. After the part has recovered from one application I have made a second close to but not upon the same spot, and nearer to the summit of the projection. Again and again I have repeated this operation, acting upon the more prominent part, until a considerable or perfect reduction of the staphyloma has been accomplished; and this has enabled me, in a few cases, to form an artificial pupil, subsequently, of much more utility to the patient. I prefer the hydrate of potash, unless the projection be very small; for its use is followed by a much larger deposit of fibrine than results from the nitrate of silver." (p. 273.)

*Conical Cornea.* Mr. Tyrrell has found, contrary to the experience of most others, the local use of stimuli retard, if not prevent, further increase of conical cornea; but he has never known any diminution occur. An operation for this disease is noticed by Mr. Middlemore (vol. i. p. 538,) in the following terms: "Where the point of the conical cornea has become opaque, and vision is thereby rendered much more obscure than it would otherwise be, it has been proposed to make an artificial pupil near to the margin of the cornea, which it is said will have two important advantages, namely, removing the pupil from the opaque part of the cornea, and allowing the light to be transmitted through the least convex part of that membrane" This operation, Mr. Tyrrell tells us, he has performed with more or less advantage in seven or eight cases.

*Anatomy of the Sclerotic.* Mr. Tyrrell here gives a somewhat inaccurate estimation of the relative extent of the cornea and sclerotica. We believe that, in a section of the eye, the sclerotic forms nearer $\frac{5}{6}$ths, and the cornea $\frac{1}{6}$th of the circumference of the exterior proper tunic of the globe than about $\frac{4}{5}$ths and $\frac{1}{5}$th respectively. Of course the cornea forms a much less part, and the sclerotica a much greater part of the sphere. As to the accuracy of the assertion, that "the part of the sclerotic which immediately overlaps the cornea receives a separate and independent supply from its conjunctival covering," we may well entertain some doubt, seeing that this part is at the most $\frac{1}{20}$th of an inch broad and $\frac{1}{80}$th of an inch in mean thickness, and that it is placed at the confluence of the arteries of the conjunctiva and anterior ciliaries.

There is nothing in Mr. Tyrrell's account of the Diseases of the Sclerotic requiring particular notice. Mr. Tyrrell describes the membrane lining the posterior chamber as the same with that lining the anterior chamber. This opinion, we think, might be justly objected to, as the two membranes have not an origin in common, and as they do not manifest exactly the same morbid phenomena. The membrane lining the posterior chamber much more readily throws out lymph and forms adhesions than that lining the anterior. Dr. von Ammon, as we mentioned in a former Number, has traced the membrane of the anterior chamber to the margin of the pupil, but never into the posterior chamber. Dr. von Ammon is also of opinion that the membrane investing the anterior surface of the iris secretes the aqueous humour, and that the lining of the cornea absorbs it. In regard to the membrane investing the posterior surface of the iris, he thinks it secretes the pigment. We would remark, however, that it is between the membrane lining the posterior chamber and the substance of the iris that the pigment of the uvea is deposited, and it is doubtless secreted by its own cells in the same way as the pigment of the membrane on the inner surface of the choroid. That the membrane of the posterior chamber secretes aqueous humour is proved by what takes places in staphyloma, in which that part of the membrane of the anterior chamber lining the cornea is gone, and that investing the surface of the iris is covered over with the tissue of cicatrice.

Mr. Tyrrell appears to include in his account of "inflammation of the aqueous membrane with deposition of fibrine, or aquo-capsulitis;" the iritis serosa of authors. Under iritis he appears to admit only iritis *parenchymatosa.* In his description of "Inflammation of the aqueous membrane producing effusion of pus without ulceration," there is no evidence adduced that the aqueous membrane is its primary seat. Indeed, Mr. Tyrrell says that it is "always in connexion with severe inflammation of many other textures of the globe." Mr. Tyrrell neglects to give a synonyme, but the disease appears to be what is usually called inflammation of the whole eye, or *panophthalmitis.*

For the affection named "Inflammation and ulceration of the aqueous membrane," Mr. Tyrrell again gives us no synonyme, and leaves us to determine whether this be an undescribed form of disease, or merely an occasional occurrence in various inflammatory affections of the eye. It appears to us to come nearest to abscess of the cornea bursting into the anterior chamber, and forming what has been called spurious hypopyon. Although Mr. Tyrrell gives no synonyme of his "Inflammation of the aqueous membrane, with increased secretion," it is evident that the disease he here describes is the inflammation of the capsule of the aqueous humour; to which Mr. Wardrop first called attention in the fourth volume of the Medico-Chirurgical Transactions, as being so remarkably benefited by the evacuation of the aqueous humour. The case related by Mr. Tyrrell at p. 327, is what is usually placed in the category of dropsy of the aqueous chambers. We think the breaking up of inflammation of the aqueous membrane into so many divisions, as Mr. Tyrrell has done, was scarcely called for by practical utility.

The "Anatomy of the Iris" is the best of Mr. Tyrrell's sketches. It is, however, not free from defects and omissions. He makes no mention of the *sinus circularis iridis* situated on the inner surface of the sclerotica where it joins the cornea. Professor Schlemm, of Berlin, was the first to point out this sinus, though Professor Arnold, of Zurich, has attempted to show that Hovius described it. This, however, is an error on the part of Professor Arnold, as what Hovius described under the name of "circulus venosus" in the eye of the ox, &c., is composed of the trunks of the choroideal veins, *vasa vorticosa,* which by their anastomoses form a circular vessel or sinus in the substance of the *choroid* about one fifth of an inch behind the canal in question. There is no such "circulus venosus" in the human eye, as the *vasa vorticosa* do not join together until where these trunks are about to pass out through the sclerotica.

*Inflammation of the Iris.* "Iritis," it is well remarked by Dr. Von Ammon, "morbus est summi quidem momenti et totius ophthalmo-pathologiæ cardo; uti enim iris in medio bulbo sita est et cum gravissimis hujus organi partibus intimum habet commercium, sic doctrina de iritide centrum occupat totius artis ophthalmiatricæ." (p. 6.) In our Nineteenth Number, in mentioning Dr. Von Ammon's divisions and subdivisions of iritis, though we remarked that there was manifested in them too much wire-drawing, and though in some of his forms of iritis the disease appears to be seated as much in other parts of the eye as the iris, we would by no means have it inferred that we differ from him in the following statement: "Pro varia vero inflammationis sede, gradu et natura, pro diversa causarum ratione, pro varia ægroti ætate, sexu et constitutione multiplices oriuntur iritidis differentiæ, quas singulas distinguere medicus potest et debet. Longissimam constituunt mutationum seriem varii gradus morbi, quæ inter symphoresin iridis simplicem et iritidem acutam jacent." (p. 8.) Mr. Tyrrell, however, though he has made what we consider some unnecessary divisions in the diseases of other parts (e. g. of the aqueous membrane), is of a very different opinion in regard to the affection under notice:

"Most modern authors," he says, "describe several varieties of this disease; but I deem such division of the subject to be of no practical utility, with the exception of a division into acute and chronic, which I shall therefore adopt. In fact I do not admit of the distinctions which have been attempted; but I consider inflammation of the iris to be the same, whatever may be its mode of origin; it may and does vary in intensity and in rapidity of progress; and these circumstances are depending more upon the condition of the constitutional power of the party affected than upon the mode of origin; a specific taint, by its influence upon the system, no doubt, in many cases modifies the local disease." (p. 337.)

We have already mentioned that Mr. Tyrrell appears to include the iritis serosa of authors in his inflammation of the aqueous membrane with deposition of fibrine, whilst Von Ammon, in describing his iritis serosa, says, "this inflammation of the serous investment of the anterior surface of the iris readily passes into keratitis serosa." This author further says his iritis serosa rheumatica may pass into kerato-iritis rheumatica and also into iritis parenchymatosa. Under the head of iritis, Mr. Tyrrell appears to describe iritis parenchymatosa only. We shall, however, find, by and bye, that what Von Ammon calls uveitis also finds a place; so that Mr. Tyrrell does not in reality differ so much as he would appear to do.

*Treatment.* We have already had occasion to mention how sparing Mr. Tyrrell is in the recommendation of bloodletting in the diseases of the eye, and how liberal he is in the employment of whatever is calculated to keep up the strength, as if the subjects of diseases of the eye were always peculiarly weak, and as if abstraction of blood, notwithstanding some degree of general weakness, were not occasionally called for by the importance of the organ at stake. Mr. Tyrrell very properly praises the mercurial treatment in iritis; but in contrasting it with simple depletion, he says nothing of the combination of the two kinds. In support of his views, he adduces cases in which depletion had already been employed, and that in an indiscriminate manner, before he commenced his mercurial treatment, which proved so efficacious. But this is hardly a fair way of discussing the subject. The fact is, there may be a few cases of iritis in which abstraction of blood is not required or may be dispensed with; but in most it will promote, in a very striking manner, the beneficial action of the mercury, frequently eliciting the action when this does not manifest itself, and render less of the mineral necessary. Abstraction of blood, followed up by a diaphoretic dose of calomel and opium or Dover's powder, relieves the distressing circumorbital pain. On this Mr. Tyrrell dwells very little, and for its relief is contented with recommending inunction of mercurial ointment with opium, which too often proves a very imperfect anodyne, no better, as a patient, himself a medical man, lately remarked to us, than so much water.

*Traumatic Iritis.* Mr. Tyrrell occupies most of his chapter on "Iritis from injury" with two cases, in which a cyst, of the size of a small pea, and glistening like tendon, had formed in connexion with the iris. The subject of the first case was a boy, an apprentice to a blacksmith. The cyst appeared some months after severe inflammation, produced by a small particle of hot iron, which penetrated the cornea and lodged in the iris. A fair and beautiful girl, about nine years of age, was the subject of the other case, in which the disease occurred a few months after inflammation brought on by the eye being struck with some bearded corn. The first case was treated by Mr. Scott, the other by Mr. Tyrrell. In both the cyst was removed. Mr. Scott's patient, Mr. Tyrrell believes, "did not retain useful vision afterwards." (p. 369.) In Mr. Tyrrell's own case iritis came on, to which was soon joined sympathetic iritis in the other eye. After a long persistence in the mercurial treatment, the inflammation was stopped; the eye secondarily affected recovered; but that on which the operation had been performed retained the power to perceive large objects only. (p. 372.)

The opening of the cornea, in these cases, the drawing out the cyst and part of the iris to which it was attached, and then the cutting off a portion of the iris and the cyst with a pair of fine scissors; and all this in scrofulous children whose eyes had but recently suffered from inflammation, appears to us, to say the least, treatment of very doubtful propriety, and which, we think, contrasts rather unfavorably with that adopted by Dr. Mackenzie in the following somewhat similar case:

"A lady was affected with considerable pain in one of her eyes, which presented the appearance of a small vesicle pushing into the anterior chamber from under the ciliary margin of the iris behind the lower edge of the cornea. The vesicle gradually increased, separating the iris more and more from the choroid, and the pain became severe. I punctured the vesicle or encysted tumour with the iris knife through the cornea. A minute quantity of fluid was discharged from the cyst, which immediately contracted so much that it was no longer visible. The pain was removed. The wound made in the cyst healed, it filled again with fluid, and again appeared in its former situation, but larger than before. I punctured it a second and a third time at intervals of six and eight weeks. After the third puncture it did not fill again. The iris returned to its natural place; the pain ceased entirely; and vision was preserved." (Treatise, p. 604.)

Under the head of Compound Ophthalmiæ, Mr. Tyrrell instances two kinds which are common, and which require special consideration, namely, inflammation of the conjunctiva and sclerotic or catarrho-rheumatic ophthalmia, and inflammation of the sclerotic and iris.

We have already mentioned that we regard the affections described by Mr. Tyrrell in a former part of his work, under the name of "acute inflammation of the cornea with suppuration" and "inflammation of the cornea with vesication," as merely forms of catarrho-rheumatic ophthalmia. What Mr. Tyrrell describes under this name is partly the "gonorrhœal inflammation of the external tunics and iris of Mr. Lawrence, and partly a milder form of common catarrho-rheumatic ophthalmia. The only peculiarity in his description is, that he says (p. 379) that, in what he calls catarrho-rheumatic ophthalmia, "it is very rare to find mischief to the cornea by ulceration or sloughing." For this, as regards ulceration, we can account only by supposing that Mr. Tyrrell refers the case, when ulceration, &c. occurs in the cornea, to one of the forms of disease above mentioned, which he considers under the head of inflammation of the cornea, but which we think are advantageously grouped together by authors under the head of catarrho-rheumatic ophthalmia.

We have in a former Number remarked that the names of the inflammations of the eye are in a great degree conventional. Let it not, therefore, be said that because affection of the cornea forms so marked a symptom of what is described by authors under the name of catarrho-rheumatic ophthalmia, we should with Mr. Tyrrell class the greater number of the cases under the head of diseases of the cornea. We say the greater number, because, as Dr. Mackenzie remarks (p. 443), "there is no ophthalmia, to which adults are exposed, in which ulcer of the cornea and onyx are so frequent as in the catarrho-rheumatic."

If we admitted Mr. Tyrrell's reasons for viewing the above forms of disease as essentially seated in the cornea to be valid, it would be equally correct to consider the inflammations of the conjunctiva we have already passed in review as more properly affections of the cornea, for, to use the words of Dr. Mackenzie, "The cornea is liable to suffer, more or less directly, in most of the ophthalmiæ already considered. Its superficial layer is apt to become vsscular and nebulous in the chronic stage of the puro-mucous ophthalmiæ; it is the seat of phlyctenulæ in scrofulous ophthalmia; and is often extensively destroyed by ulceration, in consequence of injuries and other causes. The spongy or lamellar substance of the cornea becomes infiltrated with pus, in catarrho-rheumatic ophthalmia; in certain cases it is perforated, layer after layer, by ulceration; and is sometimes almost entirely destroyed by suppuration or by sloughing." (p. 447.)

The type of Mr. Tyrrell's "*acute suppuration of the cornea*" is a traumatic inflammation—that which is occasionally met with as the result of abrasion of the surface of the cornea produced by scratches, as from the finger nails, but especially from the ears of corn, an accident to which reapers are much exposed. Traumatic inflammation in any of the textures of the eye imitates, so to speak, that which occurs in the same texture excited without any evident mechanical or chemical injury. The traumatic disease under consideration is very dangerous, and unless timely and actively treated by depletion and mercurialization, the internal textures of the eye become involved and the cornea infiltrated with pus. "The symptoms altogether," Dr. Mackenzie remarks, "bear a close resemblance to those of catarrho-rheumatic ophthalmia." (p. 223.) It is to be remembered, however, that the membrane of the aqueous humour and the iris very readily participate in the disease.

Mr. Tyrrell defines his "inflammation of the cornea with vesication" as "a partial separation of the conjunctival layer of the cornea by effusion of serous fluid." (p. 241.) Mr. Tyrrell thus views what is merely an appearance in some cases of catarrho-rheumatic ophthalmia as a separate disease. The following description of the ulceration which takes place in catarrho-rheumatic ophthalmia by Mackenzie, it will be seen, refers to the same thing as Mr. Tyrrell's *vesication*: "The ulcer is generally peculiar, in so far as it is apt to spread over the surface and rarely penetrates deeply into the substance of the cornea. It seems the result of exfoliation of a considerable portion of the corneal conjunctiva. I have seen such a portion loose and raised up apparently by the intervention of a fluid. It must be this appearance which Beer describes as a phlyctenula; but it is more extensive than a phlyctenula, and is neither so circular nor so circumscribed." (p. 443.)

The other appearances enumerated show further that the disease Mr. Tyrrell here describes is just one of the forms commonly included under catarrho-rheumatic ophthalmia; thus there is evidence of increased action in the conjunctiva and sclerotic, many of their vessels being filled with red blood; only few of the latter but very many of the former being perceptible. Farther, Mr. Tyrrell tells us that in most instances there has been a tendency to rheumatic affections.

Mr. Tyrrell says that this form of disease is to be relieved or cured by general means only, and he mentions having found the warm bath particularly serviceable. We can readily understand how nearly all kinds of local remedies might be "employed without any beneficial result;" but this we do not consider an argument against their judicious employment, after well-directed general treatment has subdued more particularly the sclerotic and corneal part of the affection. And we find Mr. Tyrrell himself recommending, under the head of catarrho-rheumatic ophthalmia, astringent applications to the conjunctiva. As to the warm bath, it will be observed that in Mr. Tyrrell's three cases it was had recourse to only after the disease of the eye had existed, and been treated for some time, it can therefore be looked upon as having acted in those instances merely as an auxiliary.

*Inflammation of the Sclerotic and Iris.* Mr. Tyrrell gives as synonymes of this, "sclero-iritis, rheumatic-iritis, arthritic-iritis," and defines it to be "inflammatory action attacking the sclerotic and iris simultaneously." Farther on (at p. 410) he says, "In sclero-iritis the affection of the iris is usually secondary, consequent on and in some measure depending upon the sclerotitis." In iritis, on the contrary, the affection of the sclerotic, he says, is secondary, being an extension of the disease from the iris. What Mr. Tyrrell describes as iritis, then, he ought to have called "irido-sclerotitis." The fact is there is generally attendant on sclerotitis a degree of iritis, and on iritis more or less sclerotitis; and the predominance of one or other has been held sufficient to give name to the disease. We see no reason for deviating from this convention, particularly as no difference of treatment is implied in the distinction.

Both in catarrho-rheumatic ophthalmia and in rheumatic iritis Mr. Tyrrell is, we think, too chary of venesection. Most practical surgeons must know, and surely Mr. Tyrrell must have had occasion often to observe, how quickly severe internal inflammation of the eye is got under by a well-timed and well-supported bleeding, how rapidly the patient rallies, and how beneficially the use of tonics comes in afterwards.

In regard to the use of tonics in rheumatic iritis we entirely coincide with the views of Dr. Mackenzie, as expressed in the following quotation from his work:

"*Cinchona* is undoubtedly a remedy of considerable utility in the treatment of rheumatic iritis. I am as much opposed, however, to the idea of trusting to it almost alone, as I am to the plan of confiding solely in the antiphlogistic and sorbefacient power of mercury in this disease to the neglect of bloodletting and other depletory means of cure. In an inflammation of so dangerous a nature as iritis we should be ready to avail ourselves of every remedy, and never allow ourselves to be beguiled into bad practice by an affectation of simplicity." (Dr. Mackenzie's Treatise, p. 465.)

*Amaurosis.* Had the arrangement Mr. Tyrrell originally intended been followed, the subject of amaurosis should have come in here; but he has preferred placing the description of the diseases of the ocular appendages at the end of the first volume, and commencing the second with amaurosis. Under the head of amaurosis, Mr. Tyrrell comprehends not only the various diseases of the nervous apparatus of the eye, but also those of the choroid, orbit, vitreous body, &c. It is not our intention here to follow Mr. Tyrrell on amaurosis, because we have already had occasion to notice (Vol. V., p. 40,) his article on the subject in the first part of the Cyclopædia of Surgery. We therefore proceed with the diseases of the eyelids.

*Entropium.* Mr. Tyrrell describes a form of entropium as depending on, besides a lax condition of the skin, a partial thickening of the conjunctiva at the point of reflexion from the lid to the globe, by which the orbital portion of the lid is pushed forwards, and the free margin consequently tilted inwards. Mr. Tyrrell appears here to have overlooked the most efficient cause of the inversion, that is, a swollen or œdematous state of the eyelid. The operation Mr. Tyrrell performs for entropium depending on contracted tarsus, is that which was employed by Ware, and consists in making a perpendicular section of the whole substance of the lid near its centre. This relieving the tension of the lid will, in some cases, be followed by a rapid removal of the inversion; but in other cases it is necessary, in order to complete the relief, to remove or destroy a part of the integument. The perpendicular section of the lid is immediately followed by a separation of the edges of the wound; and it presents an outline similar to that of the letter V; wide at the ciliary margin, and terminating in an acute point in the opposite direction. This wound is afterwards gradually filled by granulations, and very little deformity results. Mr. Tyrrell tells us he has performed this operation both on the superior and inferior palpebra; and in every case, hitherto, with perfect success. We are, therefore, inclined to recommend this operation in preference to that of Crampton, which is more complex, and it is to be confessed not always followed by perfect success. When entropium depends on a curved state of the tarsal cartilage, Mr. Tyrrell believes that little good can be done, by any method, short of the removal of the tarsus itself; or of so much of its free margin and the textures in connexion with it, as will include the cilia and the follicles from which they grow (p. 448); an opinion to which many have been driven by the frequent failure of all the nicer operations invented by Vacca, Jäger, and others.

*Ectropium.* In several severe cases of ectropium after burns, Mr. Tyrrell has adopted transplantation of a portion of skin, and in every instance with considerable advantage. (p. 456.) He gives two cases. In the second of these, two lower eyelids, a new nose, and a large extent of mouth were made, so that Mr. Tyrrell has reason to boast of having "formed many of the striking features of Master Frost's face."

Passing over Mr. Tyrrell's first five articles under the heading of "Tumours of the Palpebræ," we stop at the sixth, which is entitled "Of Encysted Tumours of the Palpebræ unconnected with the Tarsus." These tumours are frequently congenital; they contain a fatty or glairy matter, sometimes mixed with hairs, and often adhere to the periosteum. "In those cases," says Mr. Tyrrell, "in which the cyst is intimately connected with the periosteum, and this is usually indicated by the indentation of the bone, I feel little disposed to meddle with them, as I have seen very extensive mischief result." (p. 480.) Mr. Tyrrell mentions two cases in which extensive exfoliation of the bone followed unsuccessful attempts to remove the cysts. When the subject of the first case, a lady, came under Mr. Tyrrell's notice, he "could touch some extent of the dura mater, from the loss of a large part of the roof of the orbit." He adds that eventually he "succeeded in closing the wound, and, fortunately, but little deformity resulted."

*Nævus.* Mr. Tyrrell has treated nævi by injecting them with stimulating fluid; but his proceeding differs from that of Mr. Lloyd in this, that he first injects the surrounding sound cellular tissue, which becomes consolidated by the consequent inflammation. He then injects the nævus itself. Mr. Lloyd, on the contrary, injects the nævus itself alone, having previously emptied it of blood by pressure, and warns us against injecting the surrounding cellular tissue; and for this purpose he recommends pressure to be made around the nævus by means of the cover of a pill-box, with a notch on it for the passage of the point of the syringe, during the act of injecting. We thus see that what Mr. Tyrrell courts, Mr. Lloyd dreads. "A much more serious accident, however," remarks Dr. Mackenzie (p. 161), "than the injection of the cellular tissue is apt to attend this method of treating nævus, namely, the passage of some of the fluid into the veins and thence to the heart. There is strong reason to suspect that this was the cause of instant death in a child nearly two years old, in whom a nævus, situated over the angle of the jaw, was injected with diluted aqua ammoniæ."

At p. 487, Mr. Tyrrell has a chapter entitled "Of Enlargement of the Meibomian glands;" but we believe that what he describes is nothing more than a circumscribed villous state of the conjunctiva. Enlargement of the Meibomian glands is felt distinctly under the skin like a string; for being, as Dr. Zeis, of Dresden, has shown, situated in the substance of the tarsal cartilage, they become prominent as well externally as internally.

Mr. Tyrrell's next chapter is on what is called "Tinea Ciliaris" at the London Ophthalmic Hospital, elsewhere psorophthalmia, ophthalmia tarsi, &c. Having noticed the subject in connexion with Mr. Tyrrell's simple chronic ophthalmia we pass it over, as also the chapter on Trichiasis, Distichiasis, and Phtheiriasis, as containing nothing to fix the attention. We therefore come to the

*Diseases of the Lacrymal Apparatus.* Mr. Tyrrell gives but a very slight anatomical sketch of the lacrymal apparatus. His account of the lacrymal gland and its ducts is not very accurate and complete. The ducts do not open so high up as Mr. Tyrrell's description, which was certainly not taken from nature, would have them; and in regard to the gland, no mention is made of the lower mass (glandulæ congregatæ of Monro), which is situated in the substance of the upper eyelid.

Mr. Tyrrell tells us (p. 503), he has seen very few well-marked cases of disease of the lacrymal gland. At p. 505 he relates a case in which a surgeon excised the lacrymal gland by mistake along with a steatomatous tumour.

In discussing diseases of the caruncle, Mr. Tyrrell very properly warns the surgeon not to proceed hurriedly, in cases of enlargement of the body, to remove any part of it before giving his remedies a full and fair trial; "for should he do so, and the remaining part afterwards recover its proper condition, the caruncle does not present sufficient bulk to support the inner junction of the tarsi; consequently, the puncta lacrymalia become displaced, and the secretions are not properly carried away from the surface of the eye, therefore epiphora results." To this we might add, that, in puro-mucous conjunctivitis, the semilunar fold is apt to become very much enlarged; and we have seen a considerable piece of it cut away, under an impression that it was an excrescence from the conjunctiva. Small red excrescences, or polypi, grow from the caruncle. At the moment we write, we have under our eye a case in which a small red excrescence is seated, not on the caruncle, but in the depression between its upper part and the semilunar fold. Some authors speak of scirrhus primarily affecting the caruncle; but such a disease must be rare, if it occurs at all. We do not know of any recorded case. Mr. Tyrrell remarks:

"I have known the caruncle to be implicated in malignant disease, both cancerous and fungoid, such disease beginning in the lids or immediate neighbourhood; but I have never seen malignant disease commence in this body." (p. 507.)

Epiphora and stillicidium lacrymarum are used synonymously by some authors; by others, the former is employed to denote a flow of tears from the lacrymal gland, the latter the dropping of tears over the cheeks, in consequence of an impediment to their being carried off by the derivative lacrymal organs. In this latter sense, however, Mr. Tyrrell uses the word *epiphora*.

The affection Mr. Tyrrell describes under the name of contracted state of the puncta, and in which he says he has usually found most benefit derived from such general means as tend to reduce the attendant state of debility and lessen the nervous irritation, is we think rather an inordinate flow of tears from the lacrymal gland. Though he heads his chapter "*Of the contracted state of the puncta*," and tells us of their being found, on minute inspection, of so small a diameter as to be with difficulty recognized, he does not tell us what the nature of the contraction is. In relation to this subject, the following observations by Mr. Lawrence are worthy of being quoted here:

"Injuries of the lacrymal canals are mentioned as causes of stillicidium lacrymarum. [Mr. Tyrrell, it is to be remembered, uses the word epiphora for this.] My experience leads me to doubt the correctness of the statement. In a case of carcinomatous ulceration affecting the lower lid, I removed the inferior lacrymal canal; no watering of the eye ensued. I saw a gentleman with ectropium of the lower lid, consequent on a lacerated wound.....The inferior punctum, on first view, seemed obliterated; but, on close search, it was found so reduced in size as to be barely visible; from this circumstance, together with the ectropium, it was obviously incapable of absorption. There was not the slightest stillicidium." (Lawrence's Treatise, p. 702.)

Mr. Tyrrell's description of acute inflammation of the lacrymal sac is distinguished for simplicity, clearness, and brevity. The following is his etiology of chronic inflammation of this part:

"Either extension of similar disease by continuity of surface, from the conjunctival layer of the palpebræ to that of the puncta, and thence to the sac, or otherwise, the irritation of a morbid secretion, resulting from chronic disease of the palpebræ, which is conveyed through the puncta to the sac, where it creates a similar disease. It may also arise in the sac, or mucous membrane of the sac, without any previous affection of the palpebræ; but I consider such cases extremely rare, unless produced by external injury. The disease occurs at all periods of life, but is much more frequent in those of scrofulous habit than otherwise." (p. 519.)

The second of the causes enumerated above, it will be seen, is that first mentioned by Scarpa. It is rejected, and we think justly, by Lawrence and Mackenzie. The former author expresses his conviction that "the lacrymal sac and nasal duct are the original and essential seat of the disease, while the palpebral affection, when it exists, is only secondary." (Treatise, p. 708.) We quote Dr. Mackenzie's account at greater length:

"Chronic dacryocystitis is not unfrequently complicated, either locally or constitutionally. Locally, it may be connected with catarrhal inflammation of the Schneiderian membrane, or continued disorder of the Meibomian glands and conjunctiva; although, certainly, the doctrine of Scarpa, that the general cause of this disease is the absorption of puro-mucous fluid from the lids by the puncta is incorrect. It will, in many cases, be found that chronic dacryocystitis is modified by some faulty state of the general health, and often by scrofula.....In other cases, chronic inflammation of the excreting lacrymal organs appears to depend on the weakly constitution of the patient, although he be free from scrofula; and in others it is evidently kept up, and in some appears to be produced by the disordered state of the digestive organs. Smallpox, measles, and scarlet fever frequently call into action an occult scrofulous disposition, and, at the same time, give rise to the particular local disease which forms the subject of this section." (Treatise, p. 252.)

In the treatment of obstructed nasal duct, Mr. Tyrrell has tried the punctum probe, the probe or bougie from the nose, injections of water by Anel's syringe, but without any decided relief. He has also "repeatedly endeavoured to overcome the obstruction by introducing a fine *silver* tube, connected with a narrow glass cylinder capable of holding a considerable column of mercury, through one of the puncta into the sac; so as to throw the weight of the column upon the duct; but this plan has not proved of any more service than those just detailed." (p. 522.) The above, the reader will perceive, is the plan which was described by Sir William Blizard in a communication made many years ago to the Royal Society, (Philosophical Transactions for 1780; Vol. lxx., Part i., p. 239,) but we believe Sir William's instrument had a *steel** tube and

* The intelligent reader need not be informed that a *silver* tube would be incompatible with *quicksilver*.

stopcock—an instrument in fact the same as Mascagni's for injecting the lymphatics. The result of Mr. Tyrrell's experience leads him to recommend the practice which we believe is generally followed, namely, that after a fair trial of leeches, followed by astringents and stimulants, has proved unsuccessful, an operation should be performed for the purpose of introducing either a style or a tube. Mr. Tyrrell prefers the former, principally because it can be so readily removed, if irritation or inconvenience result. Mr. Tyrrell uses the old-fashioned clumsy tube, which "requires much force to fix it properly in its place; the projection just below the cup or head being of greater diameter than the duct itself: in pressing the instrument into its place, the force employed *breaks the unguis*, .......... it subsequently becomes fixed by its projection as the unguis unites, and remains hid in the sac and duct." Mr. Tyrrell tells us he has introduced above fifty of these tubes and with very excellent success. "I have only," he says, "had to remove two of them, and know of one other having been taken out by another surgeon: in two other cases the tube became obstructed; but I regularly cleared it by a punctum probe passed through one of the puncta into the tube." (p. 527.) Mr. Tyrrell does not tell us of what metal his tubes were made nor how long he continued to see his patients after the operation. Silver tubes are frequently decomposed and fall out in pieces through the nose.

Omitting, as we warned the reader we would, everything comprehended by Mr. Tyrrell under the subject of amaurosis and its congeners, we come to the morbid condition of the crystalline lens and its capsule.

*Inflammation of the Lens.* Mr. Tyrrell alludes to inflammation of the anterior wall of the capsule of the lens, and speaks of it merely as occurring in connexion with iritis generally; which shows he has never distinguished properly those cases described by Walther, and after him by Mackenzie and others under the name "Inflammation of the anterior hemisphere of the capsule," and by Dr. von Ammon under that of *Iridoperiphakitis.* The subjects of the cases, few in number, from which Mr. Tyrrell drew his description of inflammation of the lens were young. The disease appears to be of the same nature as that noticed by Mr. Lawrence (Treatise, p. 405,) in the following quotation. After mentioning the increased size of the lens, Mr. Lawrence says, "The capsulo-lenticular cataract is frequently caused by a chronic and almost insensible inflammation, or, at least, determination of blood to the eye, accompanied, not unfrequently, with symptoms of congestion in the head." What Mr. Lawrence here alludes to, we suppose, are the symptoms mentioned by Mr. Tyrrell, namely, "a sense of fulness in the organ, with uneasiness about the eyebrow or forehead, and the appearance of dark or gray muscæ, and occasional scintillations or flashing of light," and considered by him evidences of congestion in the choroid and retina.

*Cataract.* In regard to the different kinds of cataract, Mr. Tyrrell remarks, with very great truth, that the only useful divisions are such as tend to practical good. In this respect, therefore, he thinks, as concerns lenticular cataract, it is necessary to make two distinctions only, soft and hard. "This division I shall adopt," he says, "but in my description of these I shall endeavour to point out the principal varieties of the disease, and explain the modification of treatment required in each." He further says, and with great propriety, of capsular cataract, that "there is little utility in any division, further than as regards the origin or cause of the disease, which materially influences the prognosis, and requires a modification in treatment by operation." (p. 351.)

Muscæ volitantes sometimes occur before or during the formation of cataract; but, to prove that they are not owing to the disease of the lens, Mr. Tyrrell adduces the fact, that he has many times removed cataracts from patients who have complained of muscæ, and he has invariably found that the muscæ continued after the cataract had been got rid of. We may mention that Mr. Tyrrell is of opinion that the appearance of spots or muscæ, or sparks or flashes, result from an affection of the choroid or retina; he therefore recommends they should be carefully noted, as they require distinct treatment, and render the prognosis of the case, as regards the success of operation, more uncertain than when the cataract forms independent of them.

*Diagnosis.* It is well known to dissectors of the eye, that the crystalline lens, after lying a short time in water, becomes slightly opaque, presenting a peculiar mother-of-pearl colour, or a glistening appearance somewhat like tendon. The same appearance presents itself in commencing lenticular cataract. Sir David Brewster has shown that the mother-of-pearl appearance depends upon the fibrous structure of the lens. This appearance, Mr. Tyrrell says, "has been supposed by many, and is described in most works upon the subject, as indicative of opacity of the capsule," and claims to have first proved it to be in the lens; so that he has "no hesitation in declaring that it is not an indication of capsular opacity." Mr. Lawrence's description (p. 403) of the opaque spots or streaks in capsular cataract being of a *glistening*, *chalky*, or *pearly white*, affords some colouring to Mr. Tyrrell's statement, though it does not justify it; for the chalky, pearly whiteness Mr. Lawrence describes is quite different from the mother-of-pearl or tendinous appearance in the lens alluded to by Mr. Tyrrell. Dr. Mackenzie gives the same description of capsular cataract as Mr. Lawrence at p. 649 of his Treatise; but in describing lenticular cataract, says, "sometimes it has a *pearly shining appearance*, occasionally it is marked by radii from its centre towards its circumference, the lens already tending to break into such divisions as we see it fall into when left to putrefy or to undergo desiccation." (p. 648.) We would ask Mr. Tyrrell wherein this description of Dr. Mackenzie differs essentially from his own? For ourselves, we have long been perfectly aware of the seat of the glistening tendinous appearance in lenticular cataract.

At p. 363, Mr. Tyrrell notices what he considers a prevalent error in regard to the state of the lens which he describes in the following extract: "In rare instances, when the cataract commences from the circumference, and proceeds by radii towards the centre, these radii are at first confined to the posterior hemisphere of the lens." Mr. Tyrrell is quite correct in stating the supposition to be erroneous, that this state is indicative of posterior capsular opacity; but when he asserts indiscriminately that it is a prevailing opinion, we must say he lies under a mistake. "It is not an uncommon appearance," says Dr. Mackenzie (p. 648), "to see opaque striæ stretching from the circumference of the lens a short way into its substance. If such striæ run upon the back of the lens towards its centre, they seem to be situated in the vitreous humour; but this is an optical illusion." We might adduce farther, were it necessary, the same author's account of posterior capsular opacity, to show that there is nothing even in it to justify Mr. Tyrrell in his implied pretension to be considered as the corrector of the erroneous opinion he alleges to prevail.

*Operations for Cataract.* Speaking of operating for congenital cataract, Mr. Tyrrell says (p. 374), "From the experience which I have had, and which has been very great, I should recommend that the operation be performed not later than the third month, provided that the health of the child be good. I have operated in the fourth week after birth, and with perfect success."

The operation of division through the cornea was that generally preferred by Mr. Saunders in the case of infants; and, as Mr. Lawrence remarks (p. 447), "the operation of keratonyxis was well enough suited to the particular object which Mr. Saunders attempted to accomplish in congenital cataracts, namely, the formation of an opening in the opaque capsule." Mr. Tyrrell (p. 455), with others, prefers the posterior ope ration for solution in cases of congenital lenticular cataract, provided the patient be under two or three years of age. The anterior operation he adopts in cases of soft lenticular cataract, whether congenital, idio pathic, or traumatic, unaccompanied by any other disease, after the period of infancy. (p. 452.)

In connexion with the operation for congenital cataract, Mr. Tyrrell prominently sets forth the name of Mr. Saunders, as if no other one of his time but he had laboured in the same field. We, however, should be forgetful of our duty did we omit this opportunity to express the obligations this department of surgery lies under to the late Mr. Gibson, of Manchester, for the valuable contributions he made to it; but, above all, for the readiness with which he *made his experience public*, after having fully ascertained the advantages of his mode of operating. We would particularly refer the reader to Mr. Gibson's paper "On the use of the couching-needle in infants of a few months old," in the seventh volume of the Edinburgh Medical and Surgical Journal (1811), where he will find some account of the mode in which Mr. Saunders's experience was first promulgated.

Regarding the use of belladonna, for the purpose of keeping the pupil dilated, in cases whether of central cataract or opacity of the cornea opposite the pupil, Mr. Tyrrell adds to the list of cases in which the medicine has been constantly employed for years together, without any injurious effect and without losing its influence upon the iris. In one case, he relates, it has been employed for eighteen years, the application being made four or five times in the day. After this long-continued use of the narcotic, Mr. Tyrrell tells us he had an opportunity of examining the eye, the gentleman having omitted the application of it for forty-eight hours. "I then found," says Mr. Tyrrell, "the pupil of natural size, and the motions of the iris as rapid and perfect as if the belladonna had never been employed."

Mr. Tyrrell mentions (p. 381) that the cases of congenital cataract which he notices as being improved by the use of belladonna are unfavorable for operation, it having been found at the London Ophthalmic Hospital that inflammation is much more frequent after operation than in the ordinary forms of cataract. When the operation is resorted to, he recommends one eye only to be done at a time, and that with great caution and delicacy. "It is right to observe," he says, "that most of the instances in which the operation has been productive of injurious effects have occurred in patients above the age of puberty; whereas in children, little mischief has resulted under my own observation." This is not exactly in accordance with our own experience. We have frequently operated on persons about and above the age of puberty, and have found scarcely any inflammation at all ensue.

Besides the operations by extraction, by depression, and by division or solution, "I," says Mr. Tyrrell, "shall describe two others; one of *drilling* the cataract; and another in which the operations of solution and extraction, or of solution and depression, may be advantageously combined in treating the same case." After noticing various circumstances, both local and general, which may contribute to render extraction so extremely difficult and hazardous, that it should not, in Mr. Tyrrell's opinion, be attempted, he proceeds to consider the arrangements necessary to be made when the operation has been determined upon, and many of his hints will be found well worthy of attention.

Mr. Tyrrell considers it advantageous to have the patient in a recumbent posture during the operation, whilst he himself sits at the head and takes command of the upper eyelid—with the left hand if the right eye be the subject of operation, with the right hand in the contrary case; he thus uses the knife either with the right or left hand, which we, with him, consider a great advantage, especially when an intelligent assistant to take charge of the upper eyelid is not at hand.

"I have given fair trial," says Mr. Tyrrell, "to three different sections of the cornea: first, I performed that usually adopted at the time I commenced my operations on the eye—it was downwards; secondly, I made the section upwards in a great number of cases; and thirdly, I have tried an oblique section, so as to make the flap of the cornea in a direction downwards and outwards. Necessity alone would compel me to make the flap downwards again by the lower section. Of the two other plans I rather prefer the last; when prolapsus of the iris takes place after the operation to such an extent as to displace the pupil, vision is more useful and perfect if the pupil be drawn downwards and outwards, than when it is drawn upwards." (p. 400.)

This, if the assertion be correct, is a matter of great importance. The section downwards and outwards preferred by Mr. Tyrrell was that generally adopted by the first Wenzel, and has lately been pretty extensively practised by Professor Rosas, of Vienna, who uses, as Wenzel did, a double-edged knife for the purpose. The knife of Rosas is simply one of Beer's, sharpened on the back.

Mr. Tyrrell does not approve of dilating the pupil with belladonna previously to the operation—a proceeding Mr. Guthrie some time ago lauded as the essential condition of success. Our own experience would lead us to believe it to be indifferent. It is certainly of no use in facilitating the exit of the lens; as after the section of the cornea is made, and the aqueous humour has escaped, the pupil does not continue dilated, but falls into a middle state.

Mr. Tyrrell gives a chapter on the difficulties of the operation, which is well worthy of being studied by the young operator; for he must be prepared to expect the same difficulties over and over again; and a difficulty is always much less when a person is prepared for it. Mr. Tyrrell's account is true to nature. The marginal titles, " Escape of aqueous fluid," " Superior lid not properly secured," " Iris adherent to the capsule of the lens," " Lens dislocated," " Lens sinks in vitreous humour," " Protrusion of iris," " Protrusion of hyaloid membrane," &c., recall to mind untoward circumstances, often beyond the control of the operator, but, nevertheless, demanding the greatest coolness and circumspection on his part.

In regard to hemorrhage from the eye, Mr. Tyrrell says, " In some cases in which I have had to remove the displaced lens, there has been subsequent hemorrhage to such an extent as to destroy the eye." He thinks this hemorrhage arises from the artery of the posterior capsule of the lens. We doubt this for two reasons: 1st. That the artery in question is in the adult of extreme minuteness; and 2d, that hemorrhage ought to be a much more common occurrence in needle operations if this were its source. Mr. Tyrrell relates three cases of hemorrhage: in one of them he was not aware that the iris was wounded; in another he was satisfied it was not; in the third everything went on well during the operation, and the hemorrhage was discovered only after the bandages had been applied, and the patient raised from the recumbent posture. In our opinion the hemorrhage is more likely to come from the ciliary processes or the iris. From the latter we have seen it in consequence of a mere scratch with the curette. In this instance, as in all those observed by Mr. Tyrrell, the cataract had been formed for a long period.

Mr. Tyrrell does not think it proper to operate upon one eye of an elderly person whilst the other remains perfect; for it may happen that the second eye does not become affected, though this is very rare. The condition of the disease most favorable for operation, in Mr. Tyrrell's opinion, is when the cataract is perfectly formed, or nearly so, in one eye, and the vision somewhat misty in the other. Under such circumstances he extracts the perfectly-formed cataract. He objects to waiting until both cataracts are fully formed, and then operating on both eyes at once.

*Treatment after the Operation.* When there is a careful attendant, Mr. Tyrrell prefers that the patient should not go to bed, but be placed in an easy chair, or upon a sofa, in a half erect posture, when he can be more readily amused, till his usual period of rest; otherwise he is apt to sleep or doze, so much, as to prevent his having a tranquil night. If the patient have been in the habit of taking an opiate, or if he be inclined to restlessness, Mr. Tyrrell considers it prudent to administer a full dose in the evening after the operation; but remarks that this would be improper, if narcotics disagree with the patient. If symptoms of acute inflammation come on, Mr. Tyrrell recommends the abstraction of blood, generally or locally, according to the power of the patient. Besides this, he says, that " all the ordinary means to check inflammatory action must be further resorted to, if the symptoms of inflammation continue; excepting two, namely, the use of mercury, so as to affect the system, or the use of nauseating medicines. The one would prevent the union of the section of the cornea, by checking the adhesive process; the other, by occasioning vomiting, might cause the loss of vitreous humour; therefore purgatives with abstinence, and a repetition of bleeding, must be principally relied on." In regard to mercury, Mr. Tyrrell gives no case of its bad effects under the circumstances, and appears to speak rather from theory than observation. Two or three days after the operation we have put the patient under the use of mercury, with as much advantage as in an ordinary case of iritis, and certainly without the result dreaded by Mr. Tyrrell. Surely Mr. Tyrrell must have seen ulcers of the cornea healing while the mouth was affected with mercury.

Mr. Tyrrell distinguishes between the acute and subacute inflammation apt to arise after the operation. In the former, the eyelids are swollen, florid, red, and tender to the touch, with a thick yellow secretion. The conjunctiva is in a state of inflammatory chemosis. In the latter, the eyelids are rather œdematous and the secretion about the cilia thin. The conjunctiva is in a state of serous chemosis. In the subacute disease the pain is sometimes as great as in the acute; and the former is just as likely to destroy the eye as the latter, unless properly and timely cared for. In the treatment of the subacute disease, as there is great weakness and depression, Mr. Tyrrell trusts much more to nutritive diet and ordinary stimuli, than to medicinal stimuli. (p. 427.)

Mr. Tyrrell recommends that the eye should not be examined until the expiration of forty-eight hours, but thinks it better to wait seventy-two. "The reparative process," he well remarks, "is so various in different individuals, that it is impossible to determine at what period the section of the cornea may be united." (p. 437.) A good rule is, that if the eye is easy, it should not be examined for eight days.

The operation of depression, like that of extraction, is applicable to cases of hard cataract; but should, in Mr. Tyrrell's opinion, be performed only when the latter operation is impracticable or hazardous. As one of the principal circumstances to be avoided in the operation of depression, Mr. Tyrrell mentions injury to the ciliary processes; but we doubt if this can be avoided by entering the needle as he directs, so close to the junction of the cornea and sclerotica as $\frac{1}{16}$th of an inch. Mr. Tyrrell combines reclination and depression in his operation. "The result of the operation of depression," he says, "has not, under my observation, been so successful as extraction; for which reason I adopt the latter, when the case admits of it, in preference to the former." (p. 447.)

The operation of dividing the cataract in order that it may be dissolved and absorbed, is performed by introducing the needle either through the cornea or through the sclerotica; Mr. Tyrrell recommends the former mode of proceeding, "when the surgeon wishes to lacerate the anterior capsule of the lens, or simply to puncture it;" by the latter mode he thinks the division of the lens into small pieces is best effected: He calls the one the anterior, and the other the posterior needle-operation, and gives as the synonyme of the former *keratonyxis;* of the latter, *hyalonyxis.* It is worthy of remark, however, that the coiners of these words did not use them in the sense Mr. Tyrrell does. Buchhorn* was the first who introduced the term keratonyxis, but he meant by it simply

* De Keratonyxide; Halæ, 1806. De Keratonyxide, nova cataractæ aliisque oculorum morbis medendi methodo chirurgica. Magdeburg, 1810; and also Die Keratonyxis eine neue gefahrlosere Methode den grauen Staar zu operiren. Magdeburg, 1811.

*punctio corneæ*, and says expressly that the lens may, by keratonyxis, be not only divided but reclined; and that by it also an artificial pupil may be formed, whether by iridotomy or iridodialysis. As to the word *hyalonyxis*, we never saw or heard it used in the sense Mr. Tyrrell employs it. The name is not classical at all. It was introduced by an itinerant oculist of the name of Bowen, who published a small book describing a kind of operation by reclination, to which he gave the name in question. His proceeding consisted in introducing the needle through the sclerotica, at a distance of four lines from the cornea, and traversing the vitreous humour towards the posterior capsule, which he incised and then drew the lens out of its place, and pressed it down in the substance of the vitreous humour. Care was taken not to touch the anterior wall of the capsule. It may be added, that the word *sclerotonyxis* is used in an analogously extended sense, and as a counterpart of *keratonyxis*.

In the operation of division through the cornea, Mr. Tyrrell makes use of a straight needle,* merely pointed to make it penetrate with facility, but without cutting edges. He introduces it at the temporal side of the cornea near its junction with the sclerotica, the flat surface being opposed to the iris. As has been said, he limits his proceedings in this case like Conradi, who first introduced the operation, to laceration of the capsule simply. Mr. Tyrrell has observed vomiting to occur after the operation, in many cases of division through the cornea; in all cases, with one exception, when the cataract has been fluid. The vomiting generally subsided in two or three hours, but he has known it continue almost without interruption for forty-eight. "There has not been local inflammation of any importance in any of these cases."

In the operation of division through the sclerotica, or as Mr. Tyrrell calls it the posterior-needle operation, he uses a straight needle, cutting with its edges to the extent of about an eighth of an inch from the shoulder, with which, as has been mentioned, he divides the lens into small pieces. The operation he considers applicable principally to cases of congenital lenticular cataract, provided it be performed before the patient have passed the age of two or three years. "The posterior needle-operation," Mr. Tyrrell says, "should be also resorted to in cases of capsular cataract, when the lens has been removed, either by extraction or solution, resulting from operation or accident." He then goes on to prove what we believe is not denied, viz. that opaque capsule is not absorbed. By careful management, Mr. Tyrrell believes that the surgeon can restore vision in all the cases in which it is obscured by opaque capsule only, and that he may effect this with the needle. All that Mr. Tyrrell says on this subject, (pp. 461-2,) has already been very fully discussed by Mackenzie, (pp. 699-725,) but the former differs from the latter author in disapproving of extraction of, an opaque capsule. The mode of proceeding, however, which Mr. Tyrrell appears to have had in view differs from that of Mackenzie.

We now come to the modifications in the operation for cataract, viz. drilling, and a combination of solution and extraction which Mr. Tyrrell describes as new. They may be so at the scene of his labours, but they are certainly not new in the history of ophthalmic surgery. Drilling is

* Dr. Jacob's needle must be known to most of our readers.

neither more nor less than division through the cornea in cases of cataract complicated with synechia posterior and contraction of the pupil, with such additions and combinations as the circumstances of the case suggest; for instance, incision of the iris subsequently with Maunoir's scissors, in order to enlarge the pupil. The operation of drilling, Mr. Tyrrell informs us, he adopted after much careful consideration, as he found the plan followed at the Ophthalmic Hospital some years since, so unsuccessful that he dreaded to undertake the operation. The plan here alluded to appears from what Mr. Tyrrell says, (p. 464,) to have been that which is illustrated by a case recorded by Maunoir, in the Med. Chir. Trans., vol. ix. p. 287, London, 1810. Cheselden's operation for artificial pupil, by incision, combined with division or depression of the lens we have sometimes found applicable in such cases. It has the merit of being more simple than Maunoir's proceeding, and less tedious than Mr. Tyrrell's drilling.

The combination of solution and extraction is the plan which was first described by the late Mr. Gibson, of Manchester, to whom, however, Mr. Tyrrell does not allude, nor to Mr. Travers, who has written expressly on the same subject. This omission we must confess to be unfair, and by no means compensated for by Mr. Tyrrell's general acknowledgments to other authors in his dedicatory address, particularly as the language of the description is such as to lead to the supposition that the plan is original.

What Mr. Tyrrell says in his chapter entitled "Remarks on the Operations for Cataract," (p. 481,) is very judicious, but such as need not detain us, being much the same as is to be found in other authors. We cannot refrain however, from making the following extract, and adding our voice to that of Mr. Tyrrell in reprobation of the proceedings to which he alludes.

"The operation for solution is not suited to cases of hard cataract, excepting to the extent I have described: months, and even years may elapse before the aqueous humour completely dissolves the hard mass; and independent of the tedium of the cure, there is constant liability to displacement of the indurated centre, as soon as the softer circumference has become removed, and then extraction must be performed at great risk, or destructive deep-seated inflammation will ensue, and the eye become disorganized. I have seen many such unfortunate terminations to cases of hard cataract, treated by the operation of solution; and in several patients operated upon by an oculist, who unblushingly states that his operation is nearly infallible in all cases of cataract: ought not such false statements to be publicly exposed?" (p. 484.)

*Loss of the power of adapting the Eye to near and distinct Vision.* Mr. Tyrrell thinks that adaptation of the eye is owing to some change in the figure of the lens. He further believes that the power which produces the change in the lens is not in the lens itself, but is probably connected with the ciliary processes, though he does not pretend to be able to offer proof of the accuracy of such an opinion. At any rate, this opinion is an old one: (see Porterfield on the Eye.)

If, however, any change takes place in the figure of the lens it can scarcely be by means of the ciliary processes, because they have no immediate connexion with the lens. The only extrinsic structure to the action of which a change of form in the lens can be at all attributed appears to be that first described, if we are not mistaken, by Sir Everard Home, in the Philosophical Transactions, and more recently by Dr. Von

Ammon. Sir Everard Home considers the structure muscular, and thus describes it in his Croonian Lecture, published in the Philosophical Transactions, Part I., 1822, p. 77. "Between these membraneous processes (ciliary processes) there are bundles of muscular fibres of $\frac{25}{100}$ths of an inch in length, which I believe have not before been described: they originate all round from the capsule of the vitreous humour, pass forward over the edge of the lens, are attached firmly to its capsule, and there terminate." Dr. Von Ammon calls the structure orbiculus capsulo-ciliaris, and describes it as consisting of a corona of filaments extending from the inner or posterior surface of the ciliary processes to the capsule of the lens." (See Mackenzie, p. xxxi.)

One of the cases Mr. Tyrrell relates in illustration of his description appears to have something in common with those generally described under the name of mydriasis. It is not mentioned whether the subject of case 137 could converge the axes of the eyes on a near object, nor whether the defect of vision was as great when one eye only was used as when both. This case appears to be similar to one of sudden and temporary occurrence of presbyopia in a young boy lately reported (Edinburgh Med. and Surg. Journal,) with great ability by Dr. J. Hunter.

*Artificial Pupil.* Mr. Tyrrell subdivides this chapter into the following sections: 1st. Changing the natural position of the pupil. 2d. Enlargement or extension of a diminished pupil. 3d. Forming a new pupil when the natural one has been destroyed, the lens and capsule remaining perfect. 4th. Formation of a new pupil when the original opening in the iris has been destroyed, and where the crystalline has been lost. We have seen that Mr. Tyrrell recommends changing the natural position of the pupil in conical cornea; he also recommends it in cases in which the central portion of the cornea has been rendered permanently opaque. Himly, of Göttingen, we believe, was the first who introduced the practice: he opened the cornea, *prolapsed* a portion of the iris, and thus brought the pupil opposite the clear portion of the cornea. In many cases this dislocation of the pupil does not suffice; it is then necessary to excise a portion of the iris. The operative proceeding advised by Mr. Tyrrell for this purpose is that first had recourse to by Beer and afterwards by Gibson, and which is now the one most commonly employed whenever the case admits of it. The only thing in which Mr. Tyrrell is peculiar is in the use of "a fine blunt hook, with a long bend, (*Tyrrell's Hook;* see plate ix.,)" p. 500. With this hook he catches hold of the pupillary margin of the iris in order to draw it out through the corneal puncture. We have no doubt that Mr. Tyrrell's hook answers the purpose intended as well as he tells us it does; but we have also no doubt that the same thing can be effected, and with the same safety to the capsule of the lens, with a fine forceps.

In order to effect enlargement or extension of a diminished pupil, Mr. Tyrrell recommends the operation of cutting out a piece of the iris. He remarks that on operating in some of these cases, he has found that a simple fissure has resulted in attempting to draw out the iris. Under such circumstances, he has performed a second operation on a similar plan, making the opening in the cornea in a new position, so that the fissure resulting from the first might be readily extended. He adds that there may be, perhaps, rather more difficulty in avoiding injury to the capsule of the lens, in the cases under consideration, than when the iris is free; but he says he has never yet been the cause of such mischief.

Sometimes the disease, which destroys part of the cornea, and allows of escape of the aqueous fluid, prolapse of the iris, &c., is also followed by partial staphyloma. This, he justly remarks, should, if possible, be remedied before an attempt be made to extend the pupil. In illustration of this, he relates (p. 507) a very interesting and successful case, in which he first diminished the partial staphylomatous projection on the nasal side of the cornea with lunar caustic in the manner already mentioned under the head of staphyloma, and then enlarged the pupil downwards and outwards. In twenty-four hours after the operation the patient could see to read readily without the aid of a glass, and soon returned to his duties as a customhouse clerk. The right eye had been entirely destroyed by the acute purulent ophthalmia which had produced the partial staphyloma in the left.

*Of Forming a New Pupil when the natural one has been destroyed, the lens and capsule remaining perfect.* The cases Mr. Tyrrell includes under this head are those in which the whole pupillary margin of the iris is involved in a cicatrice of the cornea. He introduces his broad needle through the margin of the cornea into the anterior chamber, and pierces with it the iris close to its adhesion to the cornea, being careful not to pass the point of the instrument backward, for fear of wounding the capsule of the lens. Having thus made a very small opening in the iris with the needle, he withdraws it and then passes the blunt hook into the anterior chamber, and hooks the iris through the opening previously made in it, and gently withdrawing the instrument, usually tears the iris from the point which has been caught with the hook, "and such a quantity of the membrane can be brought through the opening in the cornea by the hook as will effect a sufficient aperture in the iris; but, sometimes only a fissure results, as in the operation before explained." Under such circumstances, Mr. Tyrrell has made, after the eye had recovered from the first operation, a second opening through the cornea, a little above the centre, and seizing the upper margin of the fissure in the iris with the hook, he has drawn it to and through the puncture of the cornea, and thus formed a triangular-shaped opening in the iris. "The principal risk in these cases," he remarks, "arises from being obliged to use a pointed instrument to effect an opening in the iris, to permit the passage of the hook, the proximity of the capsule of the lens being so close to the iris, that it is easily injured, when cataract follows." . . . . . . Mr. Tyrrell, however, says he has "very rarely inflicted injury to the lens in the operation first described." (pp. 511-12.)

*Of Formation of a New Pupil when the original opening in the Iris has been destroyed, and where the crystalline lens has been lost.* "The success of an operation for readily forming artificial pupil in all these cases," Mr. Tyrrell remarks, "depends upon the fibres of the iris being tense, so that they contract when divided." (p. 513.) He recommends Maunoir's operation by incision; but says nothing of Cheselden's, which is quite applicable in some cases and certainly more simple. In cases in which "a very small portion of the cornea has retained its healthy character, the larger part having been destroyed by slough or ulceration effecting an opening through which the lens has escaped," Mr. Tyrrell has tried two modes of making an artificial pupil: One is a very imperfect attempt at the separation of the iris from its ciliary attachment; the other, which he tells us he has found more effective and less liable to promote inflammation than the former, which after several trials he has abandoned, is described in the following words: "Using the broad needle and hook; making an opening with the former, for the passage of the latter, through the opaque cicatrix, and another in the iris, at its point of adhesion to the cicatrix; and then with the hook tearing away a portion of the iris, as before stated." (p. 517.)

We have remarked that the operation of excising a portion of the iris which Mr. Tyrrell describes is no other than that of Beer* and Gibson.† At page 43 of Mr. Gibson's work, we read: "From the principle upon which this operation is performed, it is obvious that all injury of the crystalline lens or its capsule is avoided—a most material requisite to success." (See also p. 70.) At p. 731 of Mackenzie's work it is said, in reference to remedying central opacity of the cornea by the formation of artificial pupil by excision: "It would evidently be impossible, however, to do this by incision, excision, or separation, according to the modes already described, as having been adopted by Cheselden, Wenzel, and Scarpa, without injuring the crystalline lens and thereby producing cataract. This of course must be avoided, and hence have arisen certain necessary changes in the methods of forming an artificial pupil, according to the condition of the cornea and crystalline lens." (See also p. 749.)

After all this, and after having, in our own operations, attended to the injunctions laid down in the above quotations, the reader may guess the surprise with which we read at p. 518 of Mr. Tyrrell's second volume the following, the penultimate paragraph of his chapter on artificial pupil: "Very great advantage has resulted from the introduction of the small blunt hook, inasmuch as it enables the surgeon, in many cases, to form a pupil without injury to the lens, *which was not, I believe, previously attempted.*" . . . . .

From this minute examination of Mr. Tyrrell's volumes, our readers will judge of the importance we attach to any work proceeding from a man of his long and extensive experience, and high and influential station in the profession; they will also come to the conclusion, which we have done, that in the present publication great excellence is allied with considerable imperfection. Of Mr. Tyrrell as a practical man, all that we have heard has been in favorable testimony of his qualifications. Our remarks, therefore, have reference merely to what may be judged of by the perusal of his work. And this perusal has satisfied us that experience, however great, unless aided and directed by the most accurate knowledge of the minute anatomy and physiology of the eye, and a critical acquaintance with the accumulated experience of others—however it may form a skilful practitioner—is insufficient to prepare a man to appear with advantage as an author on ophthalmic medicine and surgery.

Though we have entered so fully into detail, regarding the matter of

* Ansicht der staphylomatösen Metamorphosen des Auges, Wien, 1806.

† Practical Observations on the Formation of an Artificial Pupil in several deranged states of the Eye; to which are annexed remarks on the extraction of soft cataracts and those of the membraneous kind through a puncture in the cornea. London, 1811.

Mr. Tyrrell's work, we have as yet said nothing of the manner in which it is composed. We always feel it to be a most agreeable task to have to praise the style of medical writings, because excellence in this respect is indicative of those acquirements in literature which the public have a right to look for in members of our profession. We are sorry to say, however, that this task cannot be ours on the present occasion—not, we presume, from incapacity, but from inattention on the part of the author. It is impossible to believe that the literary faults so abounding in Mr. Tyrrell's volumes—not merely inelegancies and incorrectness of language, but mistakes as to the force and application of words, tautology, and even bad grammar,—could have any other source than that disregard to the niceties of composition which men of a practical turn of mind are too apt to fall into. This, however, is a fault and an evil, which it is the duty of those who hold the office we fill, to do their best to correct and deprecate.

---

**The first ten pages of this article having been, by mistake, printed off without revisal, some errors will be found in them, as the following: p. 58, line 13, for 'proves' read 'prove,'—line 32, for 'in' read 'on;' p. 61, last line, for 'symptom in all inflammations' read 'symptom, whenever it is present, in inflammation;' p. 62, line 36, for 'diseases' read 'disease;' p. 63, line 12, for 'last' read 'July,'—line 37, for 'diseases' read 'disease;' p. 64, line 6, after 'as' insert 'in,'—line 12, for 'proportions' read 'properties.'**

---

